# Basophilic Stippling and Ringed Sideroblasts Revealing Hidden Lead Poisoning in Refractory Microcytic Anemia

**DOI:** 10.1002/jha2.70436

**Published:** 2026-10-15

**Authors:** Huishuang Gao, Miaoke Dai, Jing Zhou, Xiang Li, Yongzhong Ning

**Affiliations:** ^1^ Department of Laboratory Medicine Beijing Chuiyangliu Hospital Beijing China

1

A man in his 60s presented with a 2‐year history of recurrent fatigue, dizziness, and severe microcytic hypochromic anemia, with a hemoglobin concentration of 56 g/L, mean corpuscular volume of 81.0 fL, and mean corpuscular hemoglobin of 25.9 pg. The anemia was refractory to empirical iron supplementation and repeated red blood cell transfusions. Baseline iron studies showed serum iron 41.50 µmol/L, ferritin 710 µg/L, and transferrin saturation 96.74%. Gastrointestinal endoscopy, renal and liver function tests, and tumor markers were unremarkable, with no known occupational metal exposure.

Peripheral blood smear review, performed according to International Council for Standardization in Haematology recommendations, revealed numerous erythrocytes with prominent coarse basophilic stippling (Figure 1) [1]. Bone marrow examination showed marked erythroid hyperplasia, accounting for 80% of nucleated cells. Prussian blue iron staining showed intracellular iron positivity in 100% of erythroblasts, with 20% ringed sideroblasts (Figure 2).

**FIGURE 1:**
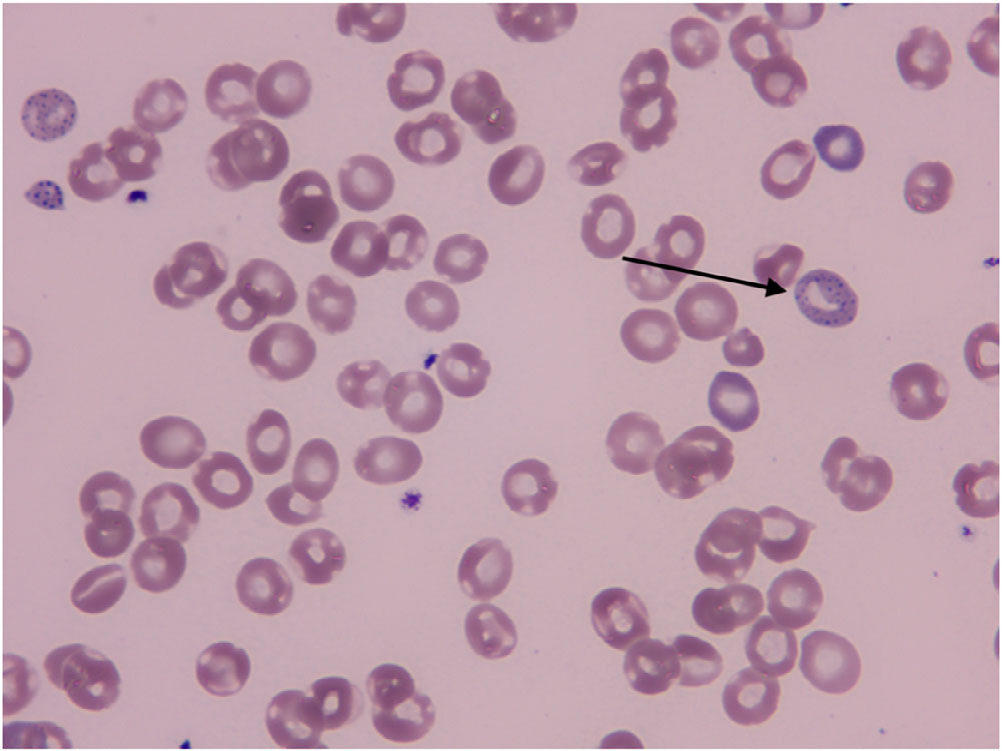
Peripheral blood smear (Wright–Giemsa stain, ×1000) showing erythrocytes with coarse basophilic stippling (arrows).

**FIGURE 2:**
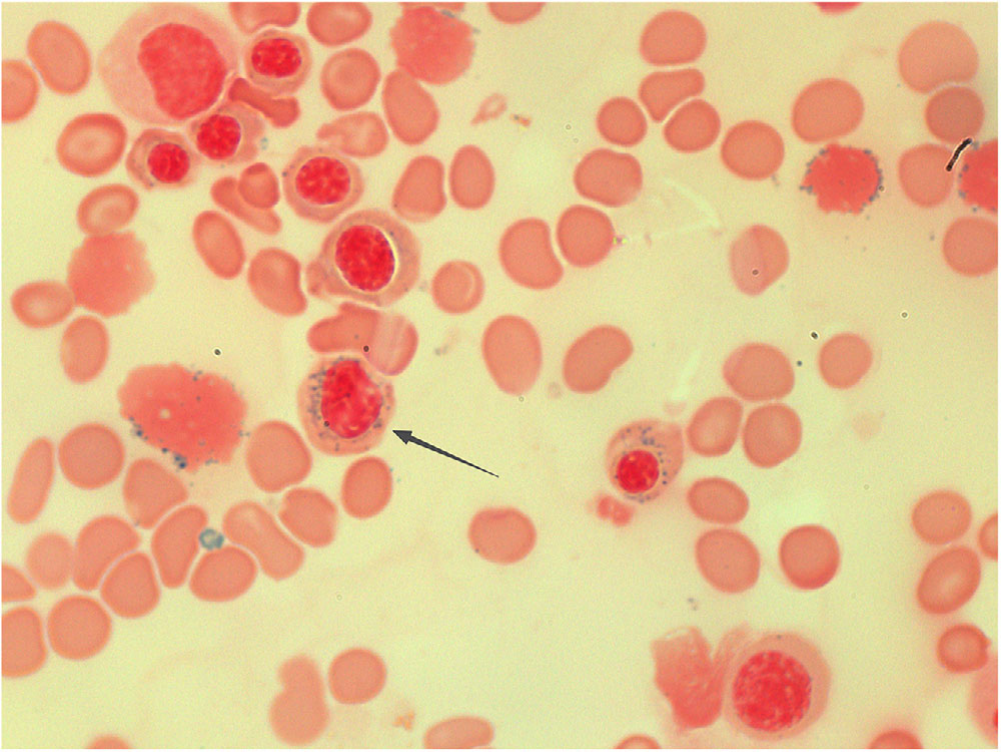
Bone marrow iron staining (Prussian blue stain, ×1000) showing ringed sideroblasts (arrow).

These concordant morphological findings prompted renewed exposure history taking, which revealed long‐term use of health supplements since 2023; the specific products were unavailable and had not been tested for lead, making them a suspected rather than confirmed source of exposure. Whole‐blood lead concentration, measured by graphite furnace atomic absorption spectrometry in K_2_EDTA‐anticoagulated blood, was markedly elevated at 828.1 µg/L (reference range, 0–100 µg/L), confirming severe lead poisoning. Mechanistically, lead inhibits δ‐aminolevulinic acid dehydratase and ferrochelatase, impairing heme synthesis and causing mitochondrial iron accumulation as ringed sideroblasts; additionally, the inhibition of pyrimidine 5′‐nucleotidase impairs ribosomal RNA degradation, accounting for coarse basophilic stippling [2].

After the suspected supplements were discontinued, the patient received five courses of specialist‐guided chelation therapy with intravenous calcium disodium EDTA (1 g once daily), consistent with recommendations for the management of severe adult lead exposure [3].

At 1 month, his hemoglobin had increased to 105 g/L and blood lead concentration had decreased to 616 µg/L. At the latest follow‐up in September 2026, the corresponding values were 111 g/L and 451 µg/L, respectively.

These images illustrate a practical laboratory hematology lesson: in adults with unexplained or refractory microcytic anemia, especially when iron therapy and transfusions are ineffective, coarse basophilic stippling should prompt consideration of lead poisoning even without occupational exposure. Concurrent ringed sideroblasts further support disturbed heme synthesis and hidden toxic exposure.

## Author Contributions

**Huishuang Gao**: investigation, data curation, formal analysis, and writing – original draft. **Miaoke Dai**: investigation, data curation, and formal analysis. **Jing Zhou**: investigation. **Xiang Li**: supervision. **Yongzhong Ning**: supervision and writing – review and editing. All authors reviewed and approved the final manuscript.

## Funding

The authors have nothing to report.

## Ethics Statement

This case image report was approved by the Ethics Committee of Beijing Chuiyangliu Hospital (Approval No. 垂杨柳伦审[2026‐035KY]号).

## Consent

Written informed consent was obtained from the patient for publication of this report and the accompanying anonymized clinical images.

## Conflicts of Interest

The authors declare no conflicts of interest.

## Data Availability

The data supporting the findings of this case report are available from the corresponding author upon reasonable request, subject to patient confidentiality and institutional policies.
